# Development of a Reference Standard of *Escherichia coli* DNA for Residual DNA Determination in China

**DOI:** 10.1371/journal.pone.0074166

**Published:** 2013-09-25

**Authors:** Lan Wang, Chunming Rao, Kai Gao, Yonghong Li, Zhihao Fu, Hua Bi, Junzhi Wang

**Affiliations:** National Institutes of Food and Drug Control, Beijing, People's Republic of China; New England Biolabs, Inc., United States of America

## Abstract

This collaborative study developed the first national *Escherichia coli* (*E. coli*) DNA reference standard for standardizing quantitative residual DNA assay methods, fluorescence dye (PicoGreen) and quantitative PCR (q-PCR), which were widely employed to measure residual DNA contents of prokaryotic-derived recombinant products. High purity of *E. coli* strain *BL21* was extracted by the cetyl triethyl ammonium bromide (CTAB)/phenol chloroform method, analyzed by UV-visible spectrophotometry and electrophoresis, diluted with tris-EDTA (TE) buffer and manually dispensed. Then, with a cooperative calibration among six laboratories, including five manufacturers and one national control laboratory, the concentration of *E. coli* DNA standard solution was determined as 96.2 μg/mL (95% C.I: 95.5–96.9 μg/mL, CV 3.4%). The candidate showed excellent stability both from accelerated degradation study and real time stability study. The applicability study showed that the *E. coli* DNA reference could reach the sensitivity of 0.781 ng/mL and 1 fg/μL, respectively, in fluorescent dye and q-PCR assay, and also had good linearity and precision. The consistency of the reference could meet the requirements of the national reference standard. As a conclusion, the candidate material was suitable to serve as a China national standard for *E. coli* residual DNA determination. The successful establishment of the *E. coli* DNA standard will facilitate the standardization of quantitative methods for testing residual host cell DNA.

## Introduction

With the rapid development of biotechnology, more and more biopharmaceuticals have been licensed to the market. The doses of biopharmaceuticals have increased from microgram to milligram or even gram for the demand of clinical treatment, and more biological products needed long-term and repeated administration. The host cells used to produce biopharmaceuticals can be sources of a range of complex, heterogeneous, and potentially unsafe impurities, and host cell DNA is among these. Much of the safety concerns associated with residual DNA in biological products lies in the possibility that host cell DNA may result in tumors or adverse reactions [1], [2]. *E. coli* expression system is favored for its advantages of low-cost, high productivity and easy operation, so it is often served as the preferred expression system of recombinant protein (e.g., recombinant cytokines, recombinant hormones, recombinant enzymes and recombinant vaccines, etc) [3]. A quality control is therefore needed to limit the residual DNA of *E. coli* derived biological products. The acceptable residual limit of DNA by World Health Organization (WHO) and European Union (EU) is up to 10 ng of residual DNA per dose [4]. The specification permitted by Food and Drug Administration (FDA) is 100 pg/dose [5]. In Pharmacopoeia of the People's Republic of China (Ch. P), the specification for DNA content is 10 ng/dose or less for bacteria-derived biologicals.

The methods used to determine the residual DNA content of biopharmaceuticals include hybridization, DNA-binding protein, q-PCR and fluorescence dye (PicoGreen), etc [6], [7], [8], [9], [10], [11]. Hybridization assay represents a semi-quantitative assay, while q-PCR and fluorescence dye give quantitative results. Quantitative assays are typically preferred instead of semi-quantitative assays because the results are considered more accurate and precise, which allows better process monitoring and release control. Generally, the laboratories or sponsors develop their own batch of *E. coli* DNA and use as the internal reference after UV absorption reading. This practice gives rise to systemic error and consequently makes the results less comparable between laboratories. In order to standardize residual DNA assay and improve inter-laboratory comparisons, we have established the first national reference standard of *E. coli* DNA suitable for two quantitative residual DNA assays (q-PCR and PicoGreen) and our results indicate that it satisfies the sensitivity and precision required for quantitative residual DNA assay. Presently, there are no public reports of similar reference standard studies for the determination of residual DNA contents of prokaryotic-derived recombinant products.

## Materials and Methods

### Preparation and quality control of *E. coli* DNA reference

*E. coli BL21* (ATCC: BAA-1025) were selected as the source materials for *E. coli* DNA standards, revived and cultured in Luria Bertani (LB) medium at 37°C for 16 h with vigorous shaking. The bacterial cells were harvested by centrifugation at 6000 g for 15 min at 4°C. The bacterial pellet was resuspended in TE buffer (pH 8.0). The suspension was used for isolating genomic DNA using CTAB/phenol-chloroform DNA extraction method. Briefly, the bacterial suspension was added 20 mg/mL protease K and 10% SDS, mixed completely, and incubated at 37°C for 1 h. The lysate was then added 5 M NaCl and CTAB/NaCl solution preheated by 65°C, incubated for 20 min at 65°C, and extracted twice with 24∶1 chloroform: isoamyl alcohol. The supernatant was precipitated by 1∶1 isopropyl alcohol, washed twice with 70% alcohol solution, and the DNA was dissolved in TE buffer. For quality control of the genomic DNA, we analyzed DNA purity by determining the ratio of spectrophotometric absorbance at 260 nm to that of 280 nm, combined with electrophoresis.

### Filling of the *E. coli* DNA reference

The extracted *BL21* DNA was diluted with sterilized TE buffer (pH 8.0) to reach a final concentration of 100 μg/mL. The solution was dispensed in 1.0 mL vials (0.1 mL each) manually and stored at −70°C.

### Concentration calibration of *E. coli* DNA reference

#### Participants

Six laboratories from China, including five manufacturers and one national control laboratories, participated in the collaborative study to calibrate the concentration of *E. coli* DNA reference using UV absorption method.

#### Study design

Precise directions were given to participants regarding sampling, dilution and storage prior to the start of the studies. In short, DNA reference was thawed, mixed well by vortex, and centrifuged at 10000 g for 2 min. A certain amount of DNA was taken and five-fold diluted with TE buffer. The optical path of spectrophotometry was set at 10 mm for the quartz cuvette, and the absorbency values at 260 nm and 280 nm wavelengths were recorded. The DNA concentration (μg/mL) was calculated by multiplying 50 with the absorbency value at 260 nm. All laboratories conducted three repeated assays for each vial, and the participants were requested to perform their assays on 5 different days. All raw data together with assay details were returned to NIFDC for analysis. All mean results are reported as unweighted geometric means with 95% confidence limits. Variability within and between laboratories has been expressed using coefficients of variation (%, CV). Participating laboratories listed in Table S1 and are referred to throughout this paper by a code number, allocated at random, and not related to the order of listing.

### Stability studies

The stability of the candidate material, *E. coli* DNA in TE buffer, was tested by accelerate thermal degradation study and the real time stability study [12]. In the accelerated thermal degradation study, three vials of the candidate material were stored at different temperatures for 4 months. Each vial, stored at −20°C, 4°C, 25°C, 37°C, was assayed for DNA content and purity by spectrophotometry and electrophoresis. Additionally, in the real time stability study, three vials of the candidate material stored at −20°C for 24 months were tested for DNA content and electrophoresis purity.

### Applicability studies of *E. coli* DNA reference

#### Fluorescence dye (PicoGreen) assay

The protocol of fluorescence dye assay for residual DNA was based on Ch.P and instructions for Quant-iT PicoGreen dsDNA kit. Briefly, *E. coli* DNA reference was serially diluted (2-fold) with TE buffer from a starting concentration of 100 ng/mL to 0.781 ng/mL, and each dilution was performed in triplicate. Then equal volumes of diluted DNA reference and the Quant-iT PicoGreen working solution were mixed and incubated for 5 min at room temperature, protected from light. After incubation, the sample fluorescence was measured using excitation wavelength 480 nm and emission wavelength 520 nm in fluorescence microplate reader and analyzed using SOFTmaxPRO software.

#### Q-PCR assay

Oligonucleotide primers against the 23S rRNA (GenBank accession number: V00331, GI: 42756) sequence of *E. coli* were used for detection of *E. coli* DNA by real-time PCR (Primer23S sense: 5′-GTC TGG AAA GGC GCG CGA-3′/Primer23S antisense: 5′-GTG TCC CGC CCT ACT CA-3′). Serial ten-fold dilutions from 10^2^ pg/μL to 1 fg/μL of *E. coli* DNA reference were prepared in TE buffer, and each dilution was performed in triplicate. PCR reaction was then carried out with the above primer pairs using the templates of serially diluted genomic DNA. PCR was performed in 7500 FAST Real-Time PCR System (Applied Biosystems, USA) using the following conditions: initial heat denaturation at 95°C for 10 min, followed by 45 cycles each of 95°C for 15 s, 60°C for 1 min. Ten microliters (10 μL) of DNA reference were amplified in a total volume of 25 μL mixture of 1×Power SYBR Green PCR Master Mix, 0.2 μM of each primer. A standard curve for quantification was generated by plotting the log of the DNA concentration of the known standard (Log Co), against the cycle number at defined point (Ct).

## Results

### Preparation and quality control of *E. coli* DNA reference

**Figure 1:**
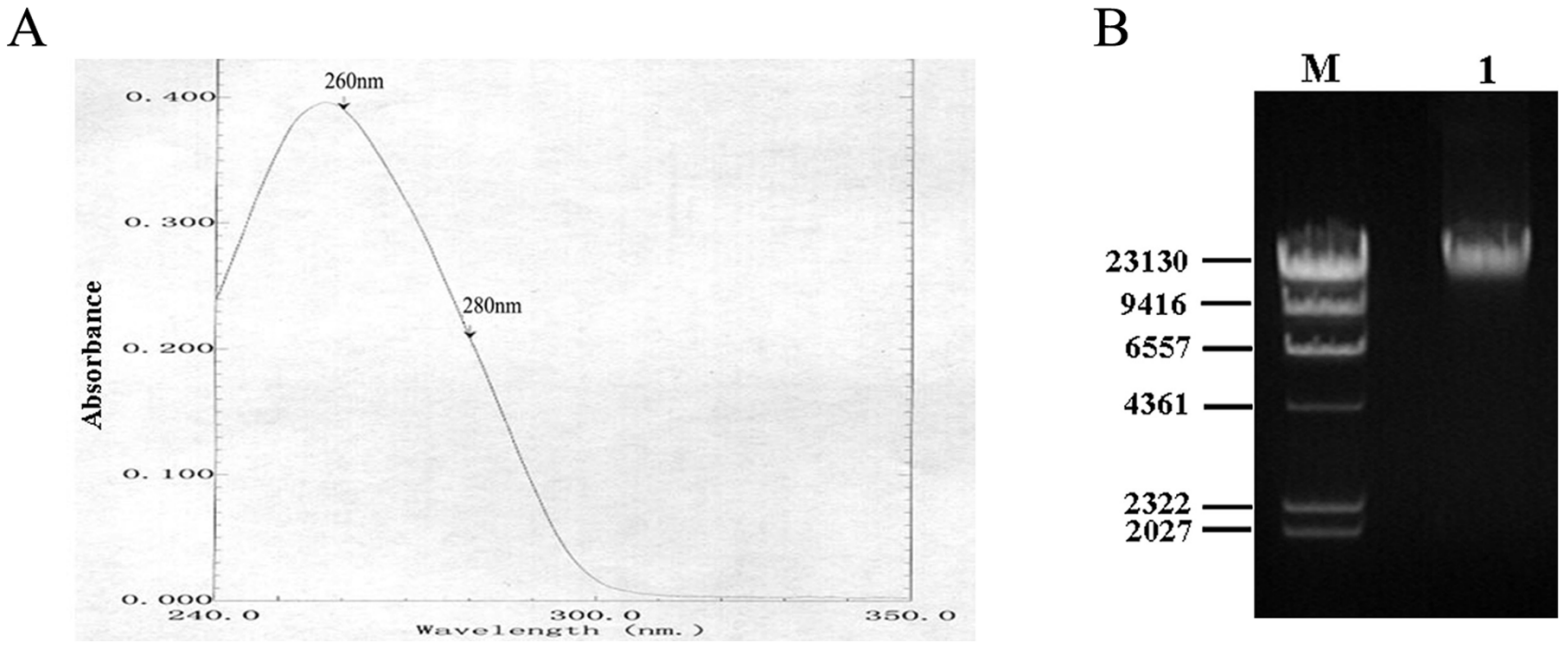
Preparation and quality control of *E. coli* DNA reference standard. (A) Analysis of *E. coli* genomic DNA with full wavelength scanning (240–350 nm). (B) Results of Agarose Gel electrophoresis for *E. coli* genomic DNA. M, λ-Hind III digest DNA marker. Lane 1, *E. coli* genomic DNA.

The genomic DNA of *BL21* was extracted and first analyzed by UV-visible spectrophotometer at wavelengths from 200 to 800 nm. As shown in Fig. 1A, the extracted DNA materials were of high quality and purity, evidenced by a strong absorption peak at about 260 nm as well as OD_260_/OD_280_ ratio of 1.8 to 1.9. In order to enlarge the observation of 260 nm absorption peak shape, Figure 1 shows a cut diagram with wavelengths from 240 nm to 350 nm. The genomic DNA of *E. coli BL21* was also analyzed by 1.0% agarose gel electrophoresis. Fig. 1B showed a single DNA band. Taken together, these results indicated that the genomic DNA prepared were of high quality and purity and satisfies the requirements of Ch. P and WHO for DNA standard samples [13].

### Concentration calibration of *E. coli* DNA reference

**Figure 2:**
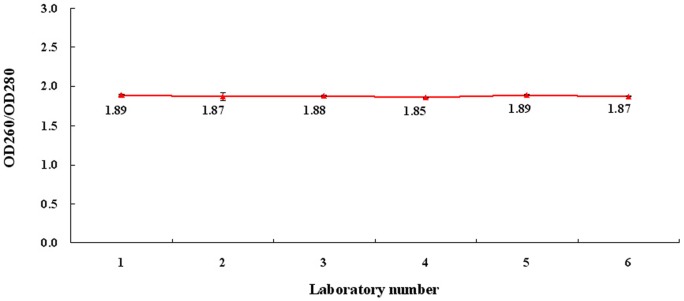
Trend of OD_260_/OD_280_ in collaborative calibration study for *E. coli* DNA reference standard.

A total of 1000 vials of *E. coli* DNA reference with a volume of 100 μL were prepared. Six certified, independent laboratories were invited to collaboratively calibrate the concentration and purity of the *E. coli* DNA. All participants performed 15 independent assays. A total of 90 results were obtained. The calibration results for *E. coli* DNA reference are summarized in Table 1. The overall geometric mean of DNA content was 96.2 μg/mL (95% confidence limits: 95.5–96.9 μg/mL, CV 3.4%, n = 90). Within-laboratory CVs ranged from 0.5% to 4.8% with an average of 2.1% indicating a small level of variability within each laboratory. The average ratio of OD260/OD280 was determined to be 1.88, well within the range of 1.8–1.9, indicating high purity of the DNA preparation (Fig. 2). In the following experiments, 96.2 μg/mLwas used for calculation and dilution purpose.

**Table 1:** The calibration results of *E. coli* DNA content by UV absorption (μg/mL).

No	Test results					Geometric mean	CV (%)	Overall geometric mean	CV (%)
	Test 1	Test 2	Test 3	Test 4	Test 5		Within Lab	95% C.I.	Overall
Lab 1	99.000	94.500	96.500	99.500	100.500	97.975	2.3		
	99.000	94.500	96.500	99.500	100.500				
	99.000	94.500	96.500	99.500	100.500				
Lab 2	92.000	93.000	93.250	94.250	93.750	93.422	1.6		
	90.750	93.250	95.000	96.250	92.750				
	91.000	92.500	95.000	95.000	93.750				
Lab 3	97.500	97.250	96.750	96.750	97.750	97.182	0.5		
	97.500	97.250	96.750	96.750	97.750				
	97.500	97.250	96.500	96.750	97.750				
Lab 4	100.925	97.450	97.950	97.575	100.800	98.832	1.7		
	100.950	97.250	98.000	97.400	100.550				
	100.950	97.350	97.750	97.350	100.425				
Lab 5	91.025	91.600	92.975	93.925	94.375	92.714	1.5		
	90.975	91.275	92.875	94.025	94.150				
	90.925	91.525	93.025	94.075	94.100				
Lab 6	88.525	101.575	99.575	97.700	98.125	97.272	4.8		
	88.675	101.500	99.975	98.125	99.025				
	88.925	101.275	100.300	98.200	99.200				
								96.2 (95.5–96.9)	3.4

Results, expressed in μg/mL, were obtained from six laboratories using UV absorption method. At least 15 independent tests were performed in each laboratory. Geometric means of each laboratory and overall geometric means with 95% confidence interval were calculated. Variability within laboratory and overall calibration were expressed as %CV.

### Stability study

**Figure 3:**
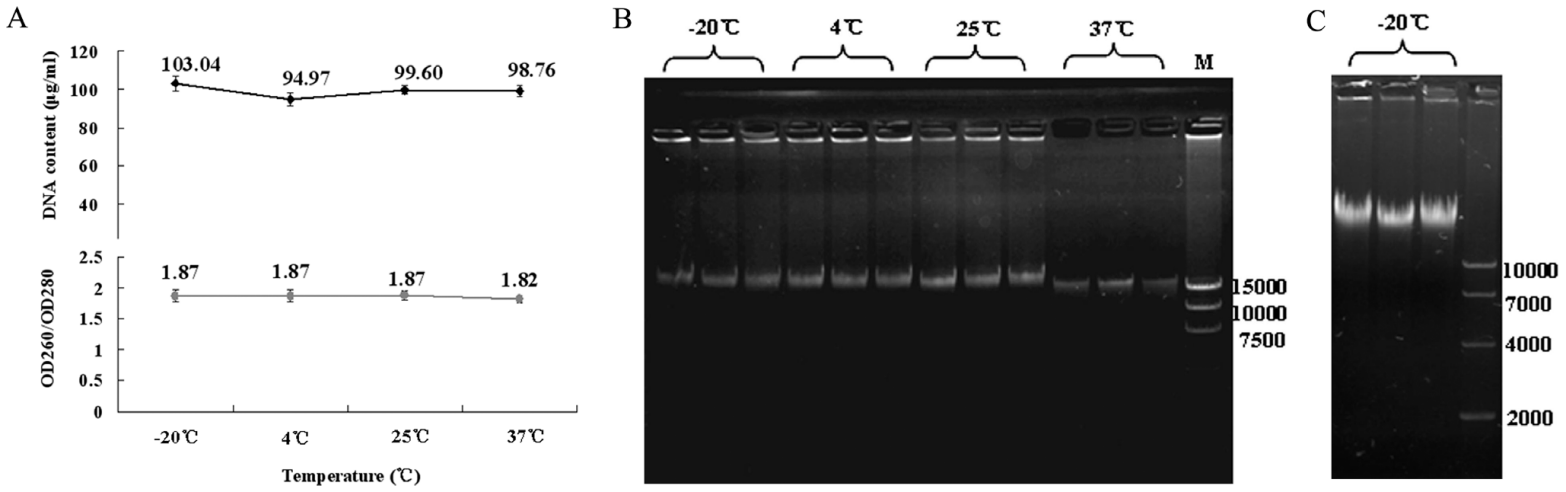
Stability of *E. coli* DNA reference standard. (A) The content and OD_260_/OD_280_ of *E. coli* DNA reference stored under four different temperatures for four months. (B) The electrophoresis of *E. coli* DNA reference stored under four different temperatures for four months. Because of high DNA content, some genomic DNA was stuck in the well. (C) The electrophoresis of *E. coli* DNA reference stored at −20°C for 24 months.

*E. coli* DNA reference stored at elevated temperatures for a period of four months was assayed for DNA content and purity by spectrophotometry and electrophoresis. As shown in Fig. 3, there was no difference observed in the DNA content and purity between the stored samples (−20°C, 4°C, 25°C, 37°C) and samples at −20°C as reference controls. In addition, real time stability study was performed using the samples stored at −20°C for 24 months. The DNA content of the samples stored at −20°C was 98.3±1.4 μg/mL, and the electrophoresis showed a single band (Fig. 3C). These results confirmed that the *E. coli* DNA reference is stable at −20°C. In generally, for liquid reference standard, the recommended storage temperature is −70°C, in order to guarantee the long-term stability of the reference standard. So as the national standard, the storage temperature of the *E. coli* DNA reference is required for −70°C.

### Applicability studies of *E. coli* DNA reference

**Figure 4:**
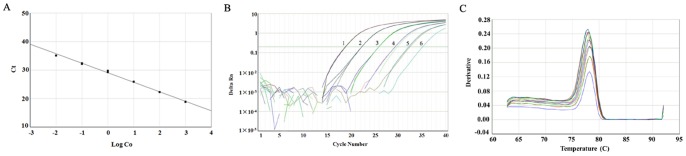
Results of real-time PCR for *E. coli* DNA reference standard. (A) Standard curve. Ct was plotted against each concentration (Log Co). Ct represents the cycle at which reporter signal was first detected. (B) Amplification curves. 1–6: 10^2^, 10, 1, 1×10^−1^, 1×10^−2^, 1×10^−3^ pg/μL. (C) Melting curves. It showed primer pair was specific for *E. coli* DNA.

Using the *E. coli* DNA reference, the PicoGreen fluorescence method had high sensitivity up to 0.781 ng/mL of DNA with good linearity in the concentration range of 0.781–100 ng/mL. Furthermore, the fluorescence CV of each concentration was less than 10% indicating good precision using the DNA reference (Table 2). By SOFTmaxPRO software, the equation of the regression line was y = 1.95x+1.65, *r* = 0.9990. For q-PCR assay, the limit of quantification (LOQ) using the DNA reference was 1 fg/μL, and the log-linear regression plot (Fig. 4A) showed a strong linear relationship (*r* = 0.9940) between the log of the DNA concentration of the known standard (Log Co) and the Ct values. The amplification curve was smooth and the dissociation curves had a single peak (Fig. 4B and C). Therefore, the standards were suitable for residual DNA assay by PicoGreen and q-PCR.

**Table 2:** Results of PicoGreen assay for *E. coli* DNA reference.

Concentration (ng/mL)	Values	Mean value	S.D.	CV (%)
100.000	164.240	164.802	3.569	2.2
	168.619			
	161.547			
50.000	83.849	86.218	2.090	2.4
	87.802			
	87.003			
25.000	44.874	45.738	0.771	1.7
	45.984			
	46.357			
12.500	24.307	23.762	0.472	2.0
	23.466			
	23.514			
6.250	11.909	12.005	0.137	1.1
	11.944			
	12.161			
3.125	6.580	6.121	0.531	8.7
	6.244			
	5.540			
1.563	3.049	3.135	0.172	5.5
	3.023			
	3.333			
0.781	1.627	1.669	0.091	5.4
	1.607			
	1.773			

### Consistency study of *E. coli* DNA reference

**Figure 5:**
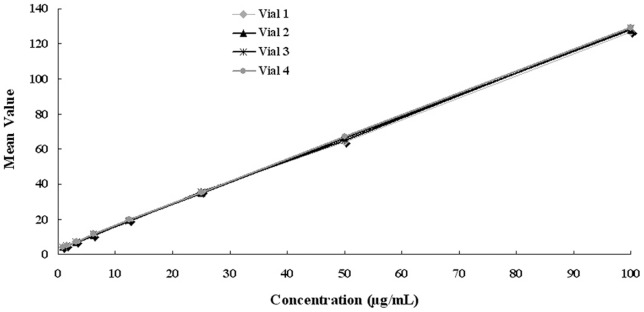
The consistency analysis of *E. coli* DNA reference standard using PicoGreen fluorescence method.

To evaluate the consistency of multiple vials of the DNA reference, the PicoGreen fluorescence method was used to test four different vials of the DNA reference. As shown in Fig. 5, four standard curves showed good consistency, and the fluorescence CVs of each concentration from four standard curves were less than 6%, which indicated the consistency of the DNA reference could meet the requirements of the national standard.

## Discussion

One can address residual DNA in biopharmaceutical processes in two ways: by validating clearance during process validation or by monitoring residual DNA levels by routine testing of the drug substance [7]. Regardless of the analytical procedures for the quantification of residual DNA, the reference material of DNA content is required. Such reference material would improve inter-laboratory comparisons and also seen as a step towards standardization of methodologies. Recently, with the expiration of patent protection for original biologics, the guidelines for biosimilar products issued or drafted by EMEA, WHO, Canada and the United States [14], [15], [16], [17] all require head to head comparative studies of quality between similar biotherapeutic product (SBP) and reference biotherapeutic product (RBP, the original brand products). The DNA reference standard also plays an important role in impurity comparative studies between SBP and RBP. *E. coli* expression system was widely used to produce biological products, so this study was initiated to develop and evaluate an *E. coli* DNA reference for quantitative residual DNA assays, including q-PCR and fluorescence assay which are widely employed in China and other countries. The most commonly used *E. coli* strains for recombinant protein production include *BL21*, *K12*, and *DH5α*, etc. Any strain of *E. coli* could be used as the source material for the *E. coli* DNA reference for the above quantitative residual DNA assays, which were both non-strain specific assays. In this study, the clearly traceable *E. coli BL21* from ATCC sources were selected as the source material for the *E. coli* DNA reference. For preparing this reference, WHO guidelines for standard materials were referred to and strictly followed [3], [18], [19], [20].

To meet the requirements for DNA standard, the following steps have been taken to ensure the quality and accuracy of *E. coli* DNA reference. First, the DNA reference solution was of high purity by a single peak at 260 nm and OD_260_/OD_280_ ratio of 1.8 to 1.9 (Fig. 1A), as well as a single band of electrophoresis (Fig. 1B). Second, the concentration of the DNA reference (96.2 μg/mL) was collaboratively calibrated by six independent, certified laboratories (Table 1 and Fig. 2). Third, results for accelerated degradation and real time stability studies indicated that the DNA reference solution was stable at −20°C (Fig. 3). In order to further control the stability of the DNA reference, we required the recommended storage temperature of the *E. coli* DNA reference was −70°C. Moreover, with high sensitivity (0.781 ng/mL and 1 fg/μL) and precision, it has satisfied the requirements for testing residual DNA of *E. coli* by PicoGreen and q-PCR (Table 2 and Fig. 4). Finally, the reference showed good consistency evaluated by PicoGreen assay (Fig. 5). Therefore, we have successfully established the DNA reference and it was adopted by the Expert Committee on Biological Standardization of P.R. China (code 2012–0026) in May 2012 as the 1st national standard for the determination of residual DNA content of *E. coli* derived products by fluorescence dye and q-PCR assay. The reference has been applied in National Control Laboratories and domestic industries in China for more than one year. It has been playing an important role in quantitatively detecting residual DNA for *E. coli* derived biologicals. Further studies are ongoing to investigate the quality of the standard throughout the life-time of the products as well as to gather stability data. It is the first time to report the *E. coli* DNA standard for quantitative residual DNA assay, which can provide clues for other similar reference product in the quality control of biologicals.

## Supporting Information

Table S1 List of participating laboratories.
(DOC)

## References

[1] Nissom PM (2007) Specific detection of residual CHO host cell DNA by real-time PCR. Biologicals 35: 211–215. 17071102 10.1016/j.biologicals.2006.09.001

[2] Knezevic I, Stacey G, Petricciani J, Sheets R (2009) Evaluation of cell substrates for the production of biologicals: Revision of WHO recommendations. Report of the WHO Study Group on Cell Substrates for the Production of Biologicals, Bethesda, USA. Biologicals 38: 162–169. 19818645 10.1016/j.biologicals.2009.08.019

[3] Gao K, Rao C, Tao L, Han C, Shi X, et al (2012) Development and calibration of a standard for the protein content of granulocyte colony-stimulating factor products. Biologicals 40: 151–157. 22296785 10.1016/j.biologicals.2011.08.013

[4] European Union (2001) Position statement on the use of tumourigenic cells of human origin for the production of biological and biotechnological medicinal products, The European Agency for the Evaluation of Medicinal Products: Evaluation of medicinal products for human use. CPMP/BWP/1143/00.

[5] U.S. Food and Drug Administration (1997) Points to consider in the manufacture and testing of monoclonal antibody products for human use. US Department of Health and Human Services, Food and Drug Administration, Center for Biologics Evaluation and Research. 10.1097/00002371-199705000-00007 9181460

[6] Kang MJ, Yu H, Kim SK, Park SR, Yang I (2011) Quantification of trace-level DNA by real-time whole genome amplification. PLoS One 6: e28661. 22174862 10.1371/journal.pone.0028661 PMC3235147

[7] Rathore AS, Sobacke SE, Kocot TJ, Morgan DR, Dufield RL, et al (2003) Analysis for residual host cell proteins and DNA in process streams of a recombinant protein product expressed in Escherichia coli cells. J Pharm Biomed Anal 32: 1199–1211. 12907264 10.1016/s0731-7085(03)00157-2

[8] Desjardins P, Conklin D (2010) NanoDrop microvolume quantitation of nucleic acids. J Vis Exp 22 (45). 10.3791/2565 PMC3346308 21189466

[9] Singer VL, Jones LJ, Yue ST, Haugland RP (1997) Characterization of PicoGreen reagent and development of a fluorescence-based solution assay for double-stranded DNA quantitation. Anal Biochem 249: 228–238. 9212875 10.1006/abio.1997.2177

[10] Kung VT, Panfili PR, Sheldon EL, King RS, Nagainis PA, et al (1990) Picogram quantitation of total DNA using DNA-binding proteins in a silicon sensor-based system. Anal Biochem 187: 220–227. 2200303 10.1016/0003-2697(90)90447-h

[11] Ji X, Lee K, DiPaolo B (2002) High-sensitivity hybridization assay for quantitation of residual E. coli DNA. Biotechniques 32: 1162–1167. 12019790 10.2144/02325dd06

[12] Campbell PJ (1974) International biological standards and reference preparations. I. Preparation and presentation of materials to serve as standards and reference preparations. J Biol Stand 2: 249–258. 4459394 10.1016/0092-1157(74)90033-x

[13] WHO Expert Committee on Biological Standardization (2006) World Health Organ Tech Rep Ser 932: v–vi, 1–137. 17274191

[14] European Medicine Evaluation Agency (2006) Guideline on similar biological medicinal products containing biotechnology-derived proteins as active substance: quality issues. pp. 5–7.

[15] WHO website (2013) Guidelines on evaluation of similar biotherapeutic products (SBP). WHO expert committee on biological standardization, 19–23 October 2009. Available: http://apps.who.int/medicinedocs/documents/s19941en/s19941en.pdf. Accessed 2013 Aug 6.

[16] Health Canada website (2013) Guidance for sponsors: Information and submission requirement for subsequent entry biologics (SEBs). 7–11. Available: http://www.hc-sc.gc.ca/dhp-mps/alt_formats/pdf/brgtherap/applic-demande/guides/seb-pbu/seb-pbu-2010-eng.pdf. Accessed 2013 Aug 6.

[17] U.S. Food and Drug Administration website (2013) Quality Considerations in Demonstrating Biosimilarity to a Reference Protein Product. 10–15. Available: http://www.fda.gov/downloads/Drugs/GuidanceCompliance RegulatoryInformation/Guidances/UCM291134.pdf. Accessed 2013 Aug 6.

[18] Niimi S, Oshizawa T, Naotsuka M, Ohba S, Yokozawa A, et al (2002) Establishment of a standard assay method for human thrombomodulin and determination of the activity of the Japanese reference standard. Biologicals 30: 69–76. 11846431 10.1006/biol.2001.0318

[19] Kline MC, Duewer DL, Travis JC, Smith MV, Redman JW, et al (2009) Production and certification of NIST Standard Reference Material 2372 Human DNA Quantitation Standard. Anal Bioanal Chem 394: 1183–1192. 19377837 10.1007/s00216-009-2782-0

[20] Cao S, Dong G, Tang J, Li J, Liu J, et al (2012) Development of a Vero cell DNA reference standard for residual DNA measurement in China. Hum Vaccin Immunother 9: 1–7. 10.4161/hv.22699 PMC3859766 23291952

