# Supplementary material for: Development of a Reference Standard of Escherichia coli DNA for Residual DNA Determination in China
Source: PLoS One. 2013 Sep 25;8(9):e74166. doi: 10.1371/journal.pone.0074166 (PMC3783418; doi:10.1371/journal.pone.0074166)
Supplement: Table S1 — List of participating laboratories. (DOC) [file pone.0074166.s001.doc]

**Supporting information**

**Table S1** List of participating laboratories

| Laboratories | Operator | Address |
| --- | --- | --- |
| Shanghai CP Guojian Pharmaceutical Co., Ltd | Dr Zhihui Zhai | No. 399 Libing Road, Zhangjiang Hi-tech Park, Shanghai, PRC, 201203 |
| Shanghai Institute of Biological Products | Dr Jiajun Yu | No. 1262 Yananxi Road, Shanghai, PRC, 200052 |
| Shenyang Sunshine Pharmaceutical Co., Ltd | Dr Baozhu Shao | No. 3A1, Road 10, Shenyang Economy & Technology Development Zone, Shenyang, PRC, 110027 |
| Xiamen Amoytop Biotech Co., Ltd | Dr Shiye Shen | No. 330 Wengjiao Road, Xinyang Industry Zone of Haicang, Xiamen, Fujian, PRC, 361022 |
| National Institutes for food and drug control | Dr Lan Wang | No.2, Tiantan Xili, Dongcheng District, Beijing, PRC, 100050 |
| Chendu Di’ao Pharmaceutical Group Co., Ltd | Dr Jun Chen | No. 26, Chuangye Road, Gaoxin Ave., High and New Technology Development Zone, Chengdu, Sichuan, PRC,610041 |
